# Long lasting response with trabectedin monotherapy in relapsed metastatic mesenchymal chondrosarcoma

**DOI:** 10.1186/s13569-020-00138-4

**Published:** 2020-08-27

**Authors:** Ghazal Tansir, Sameer Rastogi, Adarsh Barwad, Ekta Dhamija

**Affiliations:** 1 Sarcoma Medical Oncology Clinic, All India Institute of Medical Sciences (AIIMS), New Delhi, India; 2 Department of Pathology, All India Institute of Medical Sciences, New Delhi, India; 3 Department of Radiodiagnosis, Dr. B.R.A Institute Rotary Cancer Hospital, All India Institute of Medical Sciences, New Delhi, India

**Keywords:** Mesenchymal chondrosarcoma, Translocation-related sarcoma, Trabectedin

## Abstract

**Background:**

Mesenchymal chondrosarcoma is an exceedingly rare malignancy, accounting for around 5% of all patients with chondrosarcoma. It is a translocation-related sarcoma that tends to have both local and distant recurrences. Surgery is the mainstay of treatment in localised cases however treatment of advanced cases remains a challenge. The rarity of the disease precludes dedicated clinical trials and hence guidelines for its management are not well defined. The dearth in literature makes it pertinent that the cases treated with newer therapies must be reported to contribute to existing knowledge.

**Case presentation:**

We hereby report a case of a 39-year old male without any comorbidity presenting with pelvic pain and was diagnosed as mesenchymal chondrosarcoma of the pelvis. He underwent an initial curative resection followed by a disease-free interval of 7 months. Subsequently, he was treated with pulmonary metastatectomy and local debulking surgery at time of initial relapse. He was then exposed to multiple lines of palliative chemotherapy, which limited our treatment options upon subsequent disease progression. Based on recent data, the patient was given trabectedin monotherapy as fourth line chemotherapy. He tolerated the therapy well and attained a progression-free survival of 12 months, which is an impactful figure in relapsed setting in this patient population.

**Conclusion:**

This report aims to present a comprehensive review into available and newer treatment choices for mesenchymal chondrosarcoma, and to highlight trabectedin monotherapy as a possible therapeutic option for mesenchymal chondrosarcoma in the relapsed setting.

## Background

Mesenchymal chondrosarcoma (MCS) is a high-grade translocation-related sarcoma (TRS), comprising 2–9% of all chondrosarcomas [1]. It was first described by Lichtenstein and Bernstein and characterized by the presence of chondroid tissue intermixed with undifferentiated spindle cells [2]. Along with immunohistochemical positivity of vimentin, Leu7, CD99, Sox9 [3, 4], HEY1-NCOA2 is a new fusion gene recently associated with MCS [5].

The disease has a propensity for late local recurrence and distant metastases [6], however knowledge of its natural history is still incomplete due to its rarity. Studies have found that the presence of metastases is an independent predictor of disease-related mortality [7]. Localised disease has been best managed by surgical resection with or without adjuvant chemotherapy, but data is sparse on management of advanced disease. A recent study by Kawai et al. [8] found significant reduction in progression and death with trabectedin in patients of advanced translocation related sarcomas unresponsive to standard chemotherapy. Sub-group analysis of the patients of extraskeletal myxoid chondrosarcoma (ESMC) and mesenchymal chondrosarcoma included in this study, further demonstrated benefit of trabectedin compared to best supportive care (BSC) [9]. Moreover, interest has currently piqued in discovering other targets for treatment of this disease. Multiple studies currently underway, investigating Vascular Endothelial Growth Factor (VEGF) inhibitors and immunotherapy, have produced conflicting results [10–12].

We hereby report a case of relapsed, metastatic MCS who failed multiple lines of therapy and was managed with trabectedin monotherapy in our medical oncology sarcoma clinic.

## Case presentation

**Fig. 1:**
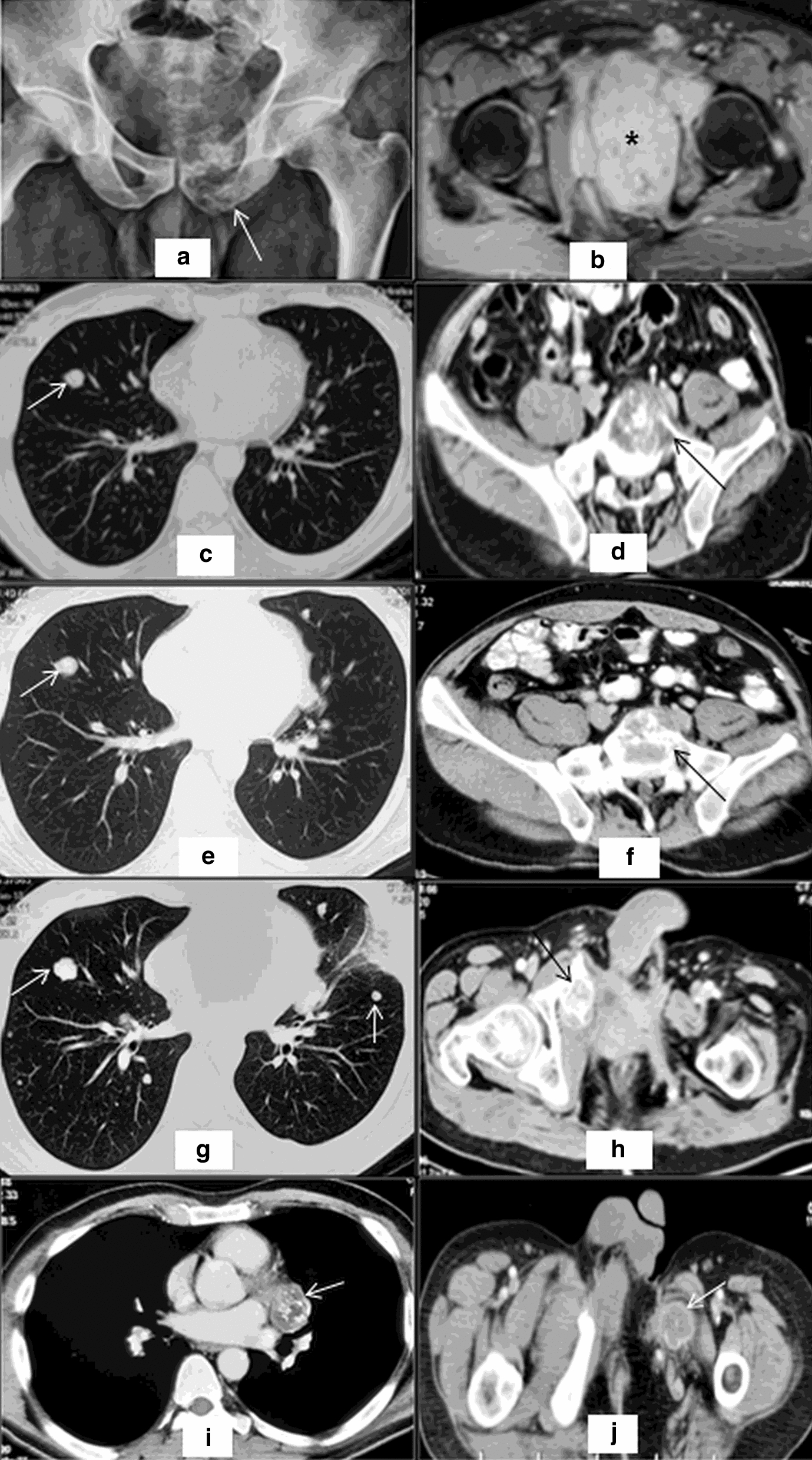
Serial imaging of the patient with pelvic chondrosarcoma. Baseline Radiograph of 2014 **a** showed permeative lytic destruction of left ischium with associated soft tissue component showing chondroid pattern of matrix mineralization. Corroborative MRI **b** showed similar findings with hyperintense mass (Asterisk) displacing the pelvic structures, not extending to hip joint. In December 2016, patient developed lung and bone metastases (arrow in **c**, **d**) after which patient was put on Trabectedin. These lesions remained stable on Trabectedin till December 2017 (**e**, **f**). CT images of January 2018 showed progression in form of appearance of new lesions in lung and bone (arrows in **g**, **h**). The disease progressed further with appearance of new lung lesions in August 2018 (arrow in **i**) and at local site in October 2018 (arrow in **j**)

A 39-year old male presented with left sided pelvic pain for 1 year. He was evaluated elsewhere where MRI pelvis was suggestive of a soft tissue lesion in left pubic bone involving the body, superior and inferior rami, extending into ischiopubic rami, ischial tuberosity and sub-articular margin of anterior pillar of acetabulum with dimension 10 × 6.5 × 10.3 cm consistent with chondrosarcoma in view of chondroid pattern of matrix mineralization (Fig. 1a, b). His distant metastatic work up was negative. He underwent type II plus type III pelvic margin-free resection with histopathologic diagnosis of mesenchymal chondrosarcoma at a different hospital with pathological staging of pT2N0M0 (Stage IB) in April 2014. A follow up scan showed normal post-operative changes with no evidence of residual disease or distant metastases and was kept on observation by treating physician. In November 2014 after disease free intevral (DFI) of 7 months, he developed asymptomatic bilateral pulmonary metastases for which pulmonary metastatectomy was planned. After a course of 3 cycles of ifosfamide-adriamycin based chemotherapy, re-assessment in January 2015 revealed marked improvement in lung nodules but there was new evidence of gross local recurrence in form of left iliac bone and acetabular soft tissue lesion with irregular destruction of residual bone; the lesion was encasing the femoral head and infiltrating adjoining soft tissue. The patient underwent repeat pelvic resection in March 2015 but pulmonary metastatecomy was deferred in view of good response of pulmonary lesions to chemotherapy. In January 2016 after progression-free survival (PFS) of 11 months, the patient was found to have ill-defined soft tissue mass at left iliac bone and left hip along with another mass 7.5 × 6.5 cm dimension, at left hemipelvis suggestive of residual disease in left iliac lesion and progression of ischial lesion and multiple bilateral lung metastases. He underwent repeat local resection in February 2016 along with palliative radiotherapy to the involved site. However, the pulmonary metastatic nodules were not amenable to surgery. 4 cycles of palliative VAC chemotherapy (vincristine, doxorubicin, cyclophosphamide-based therapy on Ewing’s Sarcoma like protocol) were given till June 2016 but his disease progressed symptomatically along with multiple sub-pleural and parenchymal pulmonary and bone metastases. He then received 3 months of oral metronomic therapy (thalidomide, celecoxib and cyclophosphamide) due to poor performance status. On improvement of his condition, he was given liposomal doxorubicin however post four cycles, imaging showed progression in the pulmonary and bone metastases (Fig. 1c, d).

Subsequent treatment with trabectedin monotherapy was started from January 2017 at dose 1.2 mg/m^2^ as 24-h continuous infusion with dexamethasone 20 mg intravenously, every 21 days. The patient tolerated the drug remarkably well without major toxicities and had improvement in clinical symptoms. The disease showed stable disease radiologically (Fig. 1e, f) on interim assessments and he went on to receive 13 cycles of trabectedin. After a PFS of 12 months, he progressed with both local and distant disease showing pulmonary and vertebral metastatic disease (Fig. 1g, h). Pazopanib was given for 6 months but there was progression in form of appearance of new lung lesions in August 2018 (Fig. 1j) followed by change of therapy to liposomal doxorubicin and ifosfamide. However, the patient progressed while on treatment (Fig. 1i, j) and shifted to best supportive care from October 2018. He succumbed to his illness in May 2019.

## Discussion

**Fig. 2:**
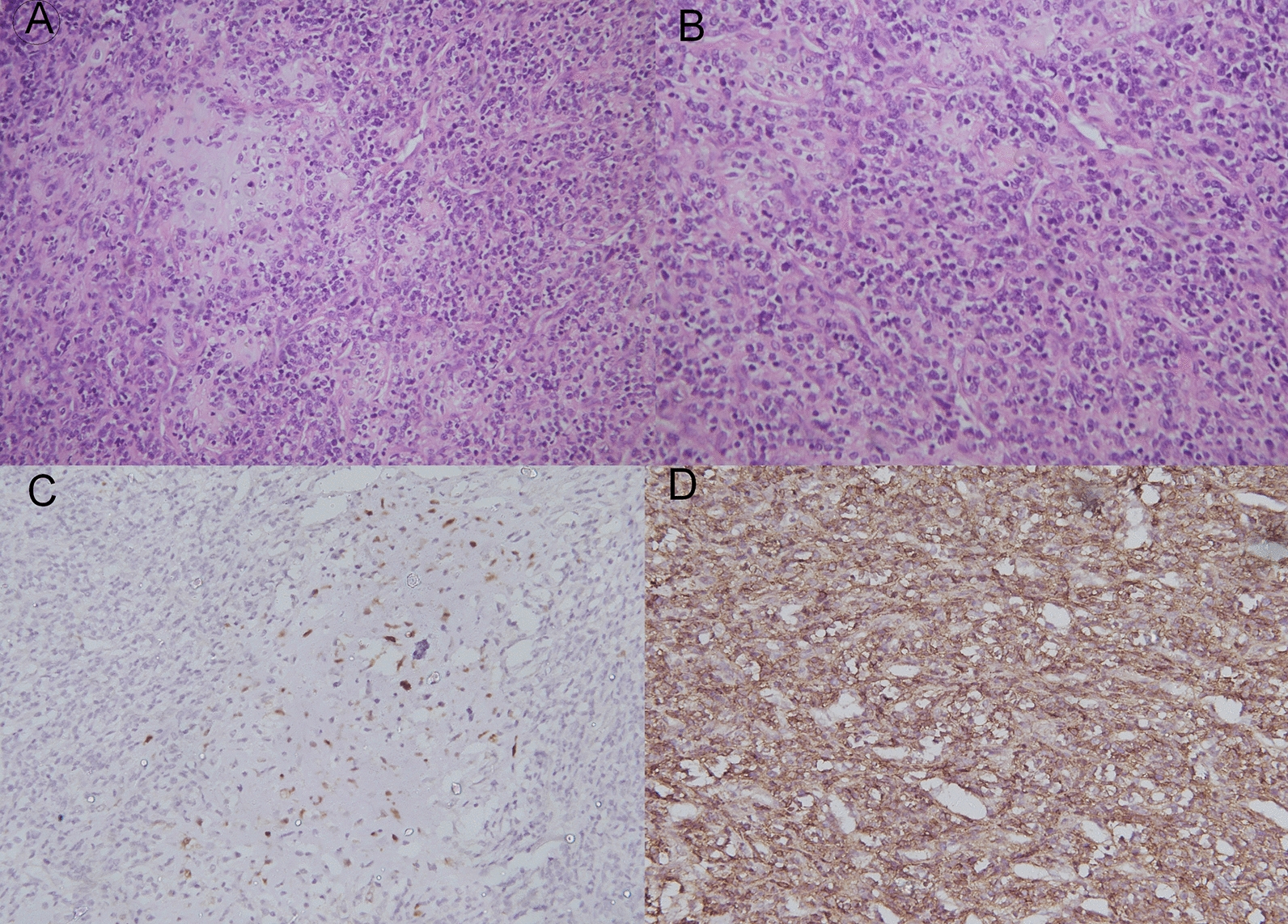
Histopathology of mesenchymal chondrosarcoma. **a** Low power picture of tumor showing malignant round cells admixed with islands of hyaline cartilage. **b** High power showing cells with high NC ratio and scanty cytoplasm admixed with lobule of hyaline cartilage showing increase cellularity. There are mitotic figures. **c** Immunostain for S100 showing nuclear positivity in Chondrocytes of hyaline cartilage. **d** Immunostain for CD99 showing membranous positivity in round cell component

Mesenchymal chondrosarcoma (MCS) is a rare entity with median age of 25 years, younger than other conventional chondrosarcomas [1]. There is a slight female preponderance among the patients. Most common primary site of origin is bone in 60% cases and soft tissue in the remainder [13]. The most common skeletal site is the axial skeleton, including craniofacial bones, ribs, ilium and vertebrae while meninges is the most frequent site of extraskeletal disease [14, 15]. Our patient was older than the median reported age for MCS with the primary location in axial skeleton, similar to other reports in the literature (Fig. 2).

Survivorship series have found 5- and 10-year overall survival (OS) of 51% and 37%, respectively [16], with axial tumors having the lowest survival rates. One of the largest studies on MCS was conducted by Nakashima et al. [17] in 1986, in which 111 new and 132 previously reported cases were studied. Survival analysis performed in 23 cases was 5- and 10-year survivals 54.6% and 27.3% respectively.

The standard treatment of localised MCS is surgical resection while chemotherapy is recommended for advanced disease. Ifosfamide and doxorubicin-based chemotherapy is standard first-line treatment however, beyond first-line there are no definite guidelines of management [18].

Trabectedin is a marine-derived antitumor agent (from marine ascidian Ecteinascidia turbinata) though now produced synthetically. It binds to minor groove of DNA, and can interfere with transcription of oncogenic fusion proteins of various types of TRS as first described in Ewing Sarcoma [19, 20]. Most common grade 3 or 4 drug-related side effects are fatigue, nausea, vomiting, neutropenia, thrombocytopenia and transient elevation of liver enzymes [21]. Although the dose of 1.5 mg/m^2^ has been recommended for use in advanced soft tissue sarcoma (STS) in literature, the dose of 1.2 mg/m^2^ has been found to have adequate antitumor activity with manageable toxicity profile in a Japanese phase I trial [22]. Hence, we used the dose of 1.2 mg/m^2^ in our patient who was already heavily pretreated, a probable reason why he had no major chemotoxicity.

Kawai et al. [8] conducted a single-arm, randomized open-label study in Japan evaluating the safety and efficacy of trabectedin in patients of advanced TRS unresponsive or intolerant to standard chemotherapy regimens. 37 patients were included in trabectedin group and 36 in BSC group. Median progression-free survival (PFS) with trabectedin was 5.6 months and 0.9 months in BSC arms, respectively. This study demonstrated significant reduction in risk of disease progression and death in advanced TRS after standard chemotherapy. Araki et al. [23] further retrospectively analyzed the patients in the BSC arm of this study who were crossed over to the trabectedin arm after disease progression. 30 patients in BSC arm received trabectedin after cross over and had median PFS of 7.3 months. Trabectedin was found to be comparable in safety and efficacy in the identical patients crossed over from BSC arm.

Morioka et al. [9] published sub-group analysis of this study in 2016. 3 mesenchymal chondrosarcoma and 2 extraskeletal myxoid chondrosarcoma patients allocated to trabectedin group were compared to 3 mesenchymal chondrosarcoma patients in BSC group. Median PFS in trabectedin group was 12.5 months versus 1.0 month in BSC. 6-month PFR was 100% in trabectedin group and the median OS was 26.4 months.

Our patient had previously failed multiple surgeries and chemotherapy lines and trabectedin served to prolong his PFS to 12 months, which is comparable with the median PFS as reported by Morioka et al. In this case of relapsed disease post three lines of chemotherapy, this PFS is especially highly significant and indicates the benefit of trabectedin in relapsed/refractory setting.

Various other novel therapies have been tried in MCS with varied outcomes. Studies have shown VEGF overproduction in chondrosarcoma cell lines, hence pazopanib has been demonstrated to produce disease stabilization in chondrosarcomas for over 6 months [24]. Though some pre-clinical studies have revealed that PD-L1 expression is absent in mesenchymal chondrosarcomas [25], research examining the role of immunotherapy in metastatic bone sarcomas is still underway. Other markers being studied as potential targets are survivin, integrin and IDH [26, 27].

As mesenchymal chondrosarcoma is a rare entity, prospective trials in this subgroup are difficult, however further studies of trabectedin in this group of tumors are warranted. Indian data too is lacking, hence efforts must be made to enroll such patients in clinical trials to establish data in our setting.

## Conclusion

In conclusion, the options for metastatic mesenchymal chondrosarcomas and other such rare soft tissue sarcomas are currently scarce. Our study demonstrates an impressive PFS of 12 months with trabectedin monotherapy used as palliative chemotherapy post-multiple lines of therapy. Hence, trabectedin can be considered as an option in advanced mesenchymal chondrosarcomas and other translocation-related sarcomas.

## Data Availability

All relevant data presented in the study are included in the article, further inquiries can be directed to the corresponding author

## Abbreviations

MCS: Mesenchymal chondrosarcoma
ESMC: Extraskeletal myxoid chondrosarcoma
VEGF: Vascular endothelial growth factor
DFS: Disease free survival
PFS: Progression free survival
OS: Overall survival
BSC: Best supportive care
STS: Soft tissue sarcoma
